# O9.4. PREDICTING SCHIZOPHRENIA: IDENTIFICATION OF MULTIMODAL MARKERS OF DISEASE THROUGH A MACHINE LEARNING APPROACH

**DOI:** 10.1093/schbul/sby015.248

**Published:** 2018-04-01

**Authors:** Linda Antonucci, Giulio Pergola, Dominic Dwyer, Silvia Torretta, Maria Teresa Attrotto, Raffaella Romano, Barbara Gelao, Rita Masellis, Antonio Rampino, Grazia Caforio, Giuseppe Blasi, Nikolaos Koutsouleris, Alessandro Bertolino

**Affiliations:** 1 University of Bari “Aldo Moro”; 2 PRONIA Consortium, Ludwig Maximilians Universitat – Munich; 3 University of Bari “Aldo Moro”, Bari University Hospital; 4 Bari University Hospital

## Abstract

**Background:**

Diagnosis of schizophrenia is based on a collection of symptoms which are heterogeneous from one patient to the other. Therefore, improving the reliability of this diagnosis is a currently unmet need. Schizophrenia risk is associated with genetic variation and with environmental factors potentially affecting neurodevelopment. Moreover, among the symptoms, cognitive abnormalities are heritable and predate its clinical onset.

Multivariate techniques can leverage the high dimensionality of data in order to study the combined effect of multiple risk factors and symptoms on clinical predictions. The aim of the current study is therefore to assess the predictability of schizophrenia diagnosis applying machine learning techniques to an ensemble of genetic, early environmental and cognitive deficits variables.

**Methods:**

442 subjects (339 healthy controls – HC – and 103 patients with schizophrenia – SCZ) were recruited for the study. Participants underwent a full neuropsychological evaluation (Modality 1, assessment of working memory, verbal fluency, intelligence quotient, attention, speed of processing and cognitive control), a broad environmental assessment (Modality 2, investigation of urbanicity, obstetric complications, developmental anomalies, socio-economic parental status and age of parents at birth) and genome-wide genotyping (Modality 3). Following published procedures, we computed individual risk scores for each of the single nucleotide polymorphisms (SNPs) associated with risk for schizophrenia in the Psychiatric Genomics Consortium (PGC) study. Data from Modalities 1, 2 and 3 entered NeuroMiner v0.998 and underwent preprocessing procedures through scaling, pruning of non-informative variables and imputation of missing values through Euclidean distance-based nearest-neighbor search. Then, these three modalities were included in a Support Vector Machine HC vs. SCZ classification algorithm, which applied decision-based data fusion strategies to integrate the individual predictions of the three modalities in a nested cross-validation framework.

**Results:**

Our cross-validated results revealed that Modality 1 (cognition) predicted schizophrenia diagnosis with the highest Balanced Accuracy (BAC, 87.3%) and that the most selected cognitive indices were intelligence quotient scores and attentional abilities. Modality 2 (environment) classified HC and SCZ with a BAC of 67.2%, and the most predictive environmental features were the parental socio-economic status, the presence of developmental anomalies during the first year of life and the age of father at birth. On the other hand, Modality 3 (genetics) predicted schizophrenia diagnosis with BAC=54,1%. The most informative SNPs were FUT9 rs117074560, TCF4 rs72934570 and STAG1 rs7432375. Decision-based fusion combining individual cognitive, environmental and genetic decision scores predicted the classification of SCZ from HC with a 78.9% BAC.

**Discussion:**

Our results using a novel machine learning approach suggest that an ensemble of cognitive, early environmental and genetic features can predict schizophrenia with significant accuracy. Our results also give key information on cognitive and environmental factors that can be targeted in early identification programs and offer novel insights about genetic loci that may be prioritized in future investigations of the pathophysiology of the disease. However, the near chance-level predictive ability of the genetic modality alone calls for the implementation and testing of more complex models of interaction between multiple risk factors.

